# (−)-Kunstleramide, a New Antioxidant and Cytotoxic Dienamide from the Bark of *Beilschmiedia kunstleri *Gamble

**DOI:** 10.3390/molecules17044197

**Published:** 2012-04-05

**Authors:** Abbas Mollataghi, A. Hamid A. Hadi, Shiau-Chuen Cheah

**Affiliations:** 1 Department of Chemistry, Faculty of Science, University of Malaya, Kuala Lumpur 50603, Malaysia; Email: abbas@siswa.um.edu.my; 2 Centre for Natural Products Research and Drug Discovery (CENAR), Department of Pharmacology, Faculty of Medicine, University of Malaya, Kuala Lumpur 50603, Malaysia; Email: cheahsc@um.edu.my; 3 School of Medicine, Faculty of Medical Sciences, UCSI University, Kuala Lumpur 56000, Malaysia

**Keywords:** *Beilschmiedia kunstleri*, lauraceae, alkaloid, dienamide, antioxidant, cytotoxicity

## Abstract

A new dienamide, (2*E*,4*E*)-7-(3',4'-dimethoxyphenyl)-*N*-ethyl-6-(*R*)-hydroxyhepta-2,4-dienamide, named (-)-kunstleramide (**1**), were isolated from the bark of *Beilschmiedia kunstleri *Gamble together with one neolignan: (+)-kunstlerone (**2**) and seven known alkaloids: (+)-nornuciferine (**3**), (-)-isocaryachine (**4**), (+)-cassythicine (**5**), (+)-laurotetanine (**6**), (+)-boldine (**7**), noratherosperminine (**8**), (+)-*N*-demethylphyllocaryptine (**9**). Their structures were established from spectroscopic techniques, most notably 1D- and 2D-NMR, UV, IR, OR, circular dichroism (CD) spectra and LCMS-IT-TOF. (-)-Kunstleramide (**1**) exhibited very poor dose-dependent inhibition of DPPH activity, with an IC_50_ value of 179.5 ± 4.4 μg/mL, but showed a moderate cytotoxic effect on MTT assays of A375, A549, HT-29, PC-3 and WRL-68 with EC_50_ values of 64.65, 44.74, 55.94, 73.87 and 70.95 µg/mL, respectively.

## 1. Introduction

**Figure 1:**
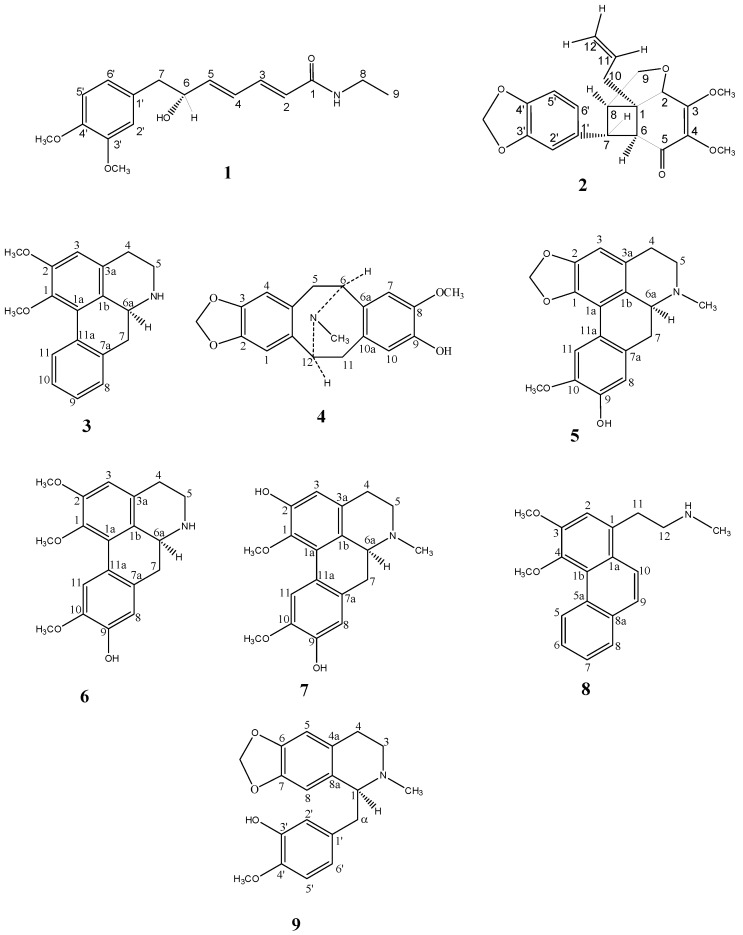
Chemical structures of compounds **1–9**.

In continuation of our research on the medicinal plants from Malaysian flora [1,2,3], we have performed a phytochemical investigation on the bark of a Malaysian Lauraceae, *Beilschmiedia kunstleri* Gamble, which has led to the isolation of a new dienamide, (2*E*,4*E*)-7-(3',4'-dimethoxyphenyl)-*N*-ethyl-6-(*R*)-hydroxyhepta-2,4-dienamide, named (−)-kunstleramide (**1**). In addition, seven known alkaloids: (+)-nornuciferine (**3**) [4], (−)-isocaryachine (**4**) [5], (+)-cassythicine (**5**) [6], (+)-laurotetanine (**6**) [7], (+)-boldine (**7**) [8], noratherosperminine (**8**) [9], (+)-*N*-demethylphyllocaryptine (**9**) [8] and one neolignan, (+)-kunstlerone (**2**) [10], were also isolated (Figure 1). This paper describes the structural elucidation, the DPPH activity with an IC_50_ value and cytotoxic effect of (−)-kunstleramide (**1**).

There are no previous reports on dienamides isolated from the Lauraceae species, although they are reported to occur in another plant species such as *Zanthoxylum ailanthoides *Sieb. and Zucc. (Rutaceae), *Piper maingayi *(Piperaceae) and *Fagara zanthoxyloides *Lam. (Rutaceae) [11,12,13,14]. Recently, we have reported one new antioxidant neolignan from the leaves of *Beilschmiedia* species [10].

## 2. Results and Discussion

(−)-Kunstleramide (**1**) was isolated as a yellowish amorphous solid. The LC-MS-IT-TOF revealed a pseudomolecular ion peak at *m/z *328.1531 [M+Na^+^], thus suggesting a molecular formula of C_17_H_23_NO_4_ (calc. 305.1525). The IR spectrum showed the presence of the bands at 1,612 and 1,659 cm^−1^, due to C=C and C=O, and 3,351 cm^−1^ due to NH and OH stretching vibrations, respectively [15,16]. The ^1^H-NMR spectrum (Table 1) established the presence of three aromatic protons at δ 6.66 (1H, *d*, *J *= 1.8 Hz, H-2'), 6.67 (1H, *dd*, *J = *8.2, 1.8 Hz, H-6') and 6.76 (1H, *d*, *J *= 8.2 Hz, H-5') and two methoxyl groups at δ 3.79 (3H, *s*, 4'-OCH_3_) and 3.81 (3H, *s*, 3'-OCH_3_).

**Table 1:** ^1^H-NMR (400 MHz) and ^13^C-NMR (100 MHz) spectral data of (−)-kunstleramide (**1**) in CDCl_3_.

Position	^1^H (δ_H_, Hz)	^13^C
C=O	-	165.8
C-2	5.74, *d* (15.1)	124.2
C-3	7.15, *dd* (15.1, 8.9)	140.0
C-4	6.26, *dd *(15.1, 11.2)	127.7
C-5	6.06, *dd* (15.1, 5.1)	142.3
C-6	4.37, m	72.6
C-7	2.66, *dd* (*J_β_* = 13.1, 5.1),2.80, *dd* (*J_α_* = 13.1, 5.1)	43.4
C-8C-9	3.30, *q* (7.4)1.10, *t* (7.4)	34.614.9
C-1'	-	129.5
C-2'	6.66, *d* (1.8)	112.6
C-3'	-	147.9
3’-OMeC-4'4'-OMe	3.81, *s*-3.79, s	55.9149.955.9
C-5'	6.76, *d* (8.2)	111.3
C-6'	6.67, *dd* (8.2, 1.8)	121.6
N-HO-H	5.43, *br s* 1.67, *br s*	--

**Figure 2:**
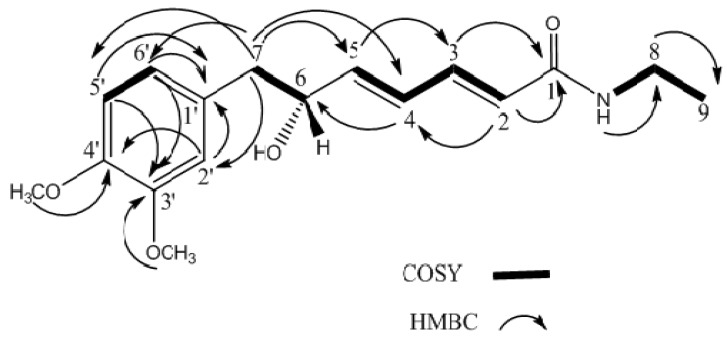
Selected 2D-NMR (HMBC and COSY) correlations of (-)-kunstleramide(**1**).

Signals representing an *N*-ethyl attached to C=O appeared as a quartet at δ 3.30 (methylene) and a triplet at δ 1.10 for the methyl group. The H-6 methine appeared as a multiplet at δ 4.37 and the H-7 methylene showed two doublet of doublets at δ 2.66 and 2.80 (*J *= 13.1 and 5.1 Hz), respectively. The olefinic proton was observed at δ 5.74 (*J *= 15.1 Hz) as a doublet for H-2. A doublet of doublets at δ 7.15 (*J *= 15.1 and 8.9 Hz) was assigned to H-3. Another two olefenic protons, H-4 and H-5 appeared at δ 6.26 (*J *= 15.1 and 11.2 Hz) as doublet of doublets and at δ 6.06 (*J *= 15.1 and 5.1 Hz) as a doublet of doublets, respectively. Therefore, the structure of compound **1** was unambiguously determined as (2*E*,4*E*), which was also confirmed by H-H COSY, H-H NOESY, and DEPT spectra [16]. ^13^C-NMR and DEPT spectra (Table 1) showed three methyls, two methylenes, eight methines, three quaternary carbons and one carbonyl. The sp^2^ carbon of C-2 bearing an ethyl amide group gave a signal at δ124.2, while the carbonyl group was observed at δ 165.8 [16]. The correlations of H-2 and H-8 with C-1, and H-N with C-8 in the HMBC spectrum further confirmed the position of ethyl amide fragment at C-1. In addition, the cross-peaks in the COSY spectrum were observed between H-2/H-3, H-3/H-4, H-4/H-5, H-5/H-6, H-6/H-7 and H-5'/H-6'. The chemical shifts of proton and carbon were shown in Table 1 and the H-H correlations and H-C connectivity are shown in Figure 2.

**Figure 3:**
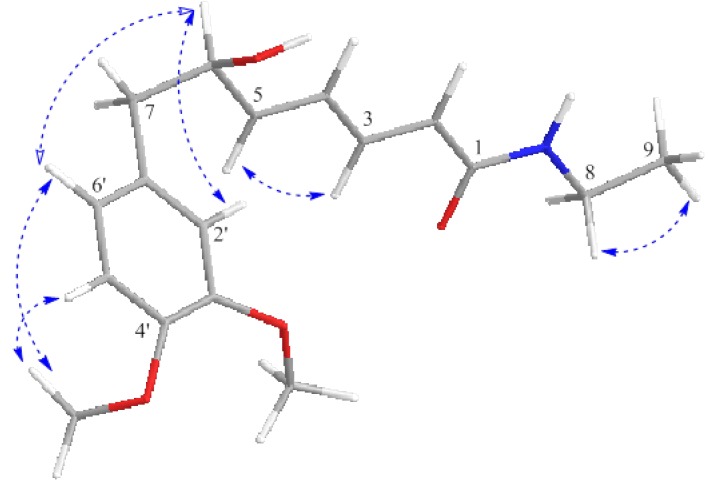
Selected NOESY correlation of (−)-kunstleramide (**1**).

The configuration of proton at C-6 was confirmed based on the analysis of NOESY spectrum. NOESY correlations were observed between H-9 (δ 1.10)/H-8 (δ 3.30), H-3 (δ 7.15)/H-5 (δ 6.06), H-7α (δ 2.80)/H-7β (δ 2.66), H-6 (δ 4.37)/6' (δ 6.67), H-2' (δ 6.66)/H-6 (δ 4.37), H-6' (δ 6.67)/4'-OCH_3 _(δ 3.79) and H-5' (δ 6.76)/4'-OCH_3 _(δ 3.79) respectively (Figure 3).

Consequently, the relative configuration of C-6 proton was *R *and the optical rotation is −15.48°. Since compound (**1**) is a chiral, there is a Cotton effect at the position of the chromophore band. The CD (circular dichroism) showed two Cotton effect curves with opposite sign around λ_max_ 262 nm separated by a Davydov splitting. Therefore, the chirality of compound **1** is due to the chiralities between the electric dipole transition moments of both chromophore (the benzene and the unsaturated amide) [17,18]. Based on the above spectroscopic data, it was confirmed that compound **1** was (2*E*,4*E*)-7-(3',4'-dimethoxyphenyl)-*N*-ethyl-6-(*R*)-hydroxyhepta-2,4-dienamide, and it was named (−)-kunstleramide.

### 2.1. Antioxidant Activity

**Figure 4:**
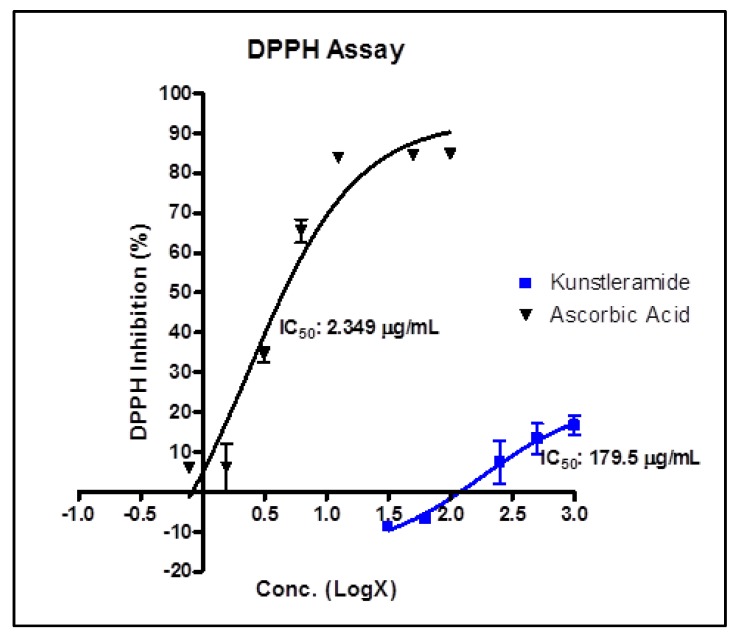
IC_50_ values of (−)-kunstleramide (**1**). AA: ascorbic acid as control. Results are means ± SD of two replicates.

The antioxidant activity of (−)-kunstleramide (**1**) was tested using a DPPH assay. Antioxidants are substances that may protect cells from the damage caused by unstable molecules known as free radicals [19]. Free radicals from oxidative stress are involved in many disorders like neurodegenerative diseases and cancer [20]. The new dienamide, (−)-kunstleramide (**1**) exhibited very poor DPPH activity, with an IC_50_ value of 179.5 ± 4.4 μg/mL compared to ascorbic acid (Figure 4). Factors such as growth conditions, stability of the specific antioxidant components, including variations in the process of extraction can influence the variations in the antioxidant activity [21].

### 2.2. Cytotoxic Activity

**Figure 5:**
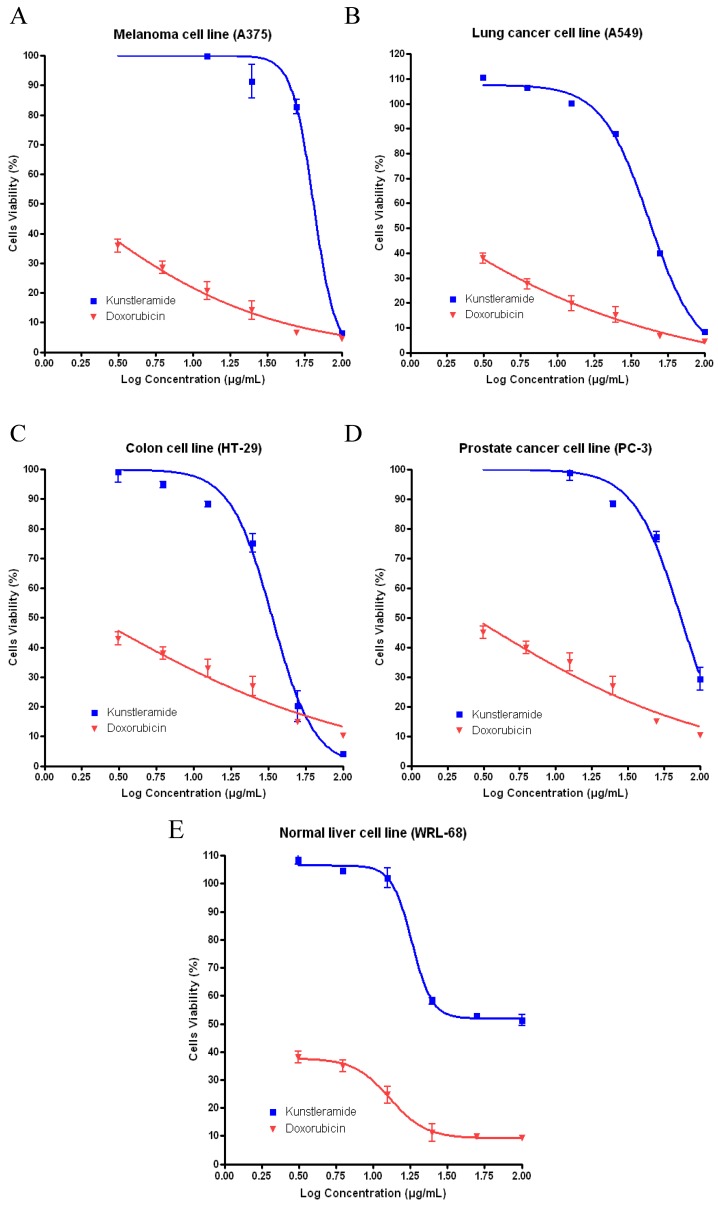
Dose-response curves (using GraphPad Prism) tested with (−)-kunstleramide (**1**) and doxorubicin (positive control) in the MTT assays towards (**A**) A375, (**B**) A549, (**C**) HT-29, (**D**) PC-3 and (**E**) WRL-68.

To evaluate the cytotoxic activity, the new compound (−)-kunstleramide (**1**) was tested with a series of different doses on A549, PC-3, A375, HT-29 and WRL-68, respectively (Figure 5). After 24 h, cell viability was determined by the MTT assay. Test agents induced cell cytotoxicity in a concentration dependent manner. These dose titration curves allowed determining EC_50_ for the test agents towards different cell lines (Table 2).

**Table 2:** Effect of compounds (−)-kunstleramide (**1**) and doxorubicin (positive control) on different cells type expressed as EC_50_ values in 24 h MTT assay. [EC_50_ ± S.D (µg/mL)].

Cell line	New Dienamide	Doxorubicin
A375	64.65	1.364
A549	44.74	1.550
HT-29	55.94	1.957
PC-3	73.87	2.125
WRL-68	70.95	1.731

**Figure 6:**
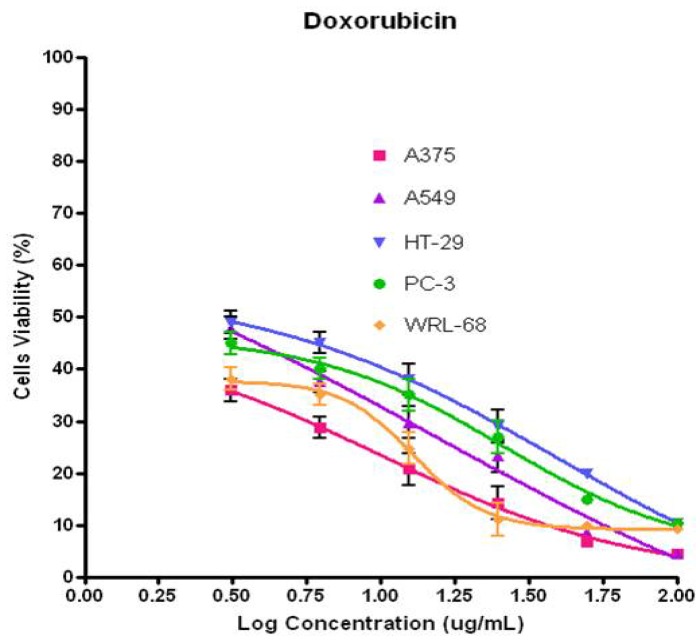
Dose-response curves (using GraphPad Prism) tested with doxorubicin (positive control) in the MTT assays towards A375, A549, HT-29, PC-3 and WRL-68.

From Figure 5, (−)-kunstleramide (**1**) showed cytotoxic effect on several of the cancer cell lines with different EC_50_ values as compared to the standard, Doxorubicin (Figure 6). This compound showed moderately cytotoxic effect. (−)-Kunstleramide (**1**) demonstrated dose-depended cytotoxic effects EC_50_ values of 64.65, 44.74, 55.94, 73.87 and 70.95 µg/mL; in A375, A549, HT-29, PC-3 and WRL-68, respectively. These results indicate that cell lines differ in their sensitivity to the same test agent, which may be determined by multiple cell type-specific signalling cascades and transcription factor activities.

To our knowledge, the cytotoxic potentials of (−)-kunstleramide (**1**) have not been examined and the underlying molecular mechanisms remain to be discovered.

## 3. Experimental

### 3.1. General

The Fourier Transform Infrared (FT-IR) spectra were obtained with CHCl_3_ (NaCl window technique) on a Perkin Elmer 2000 instrument. The ultraviolet spectra (UV) were obtained in MeOH on a Shimadzu UV-310 ultraviolet-visible spectrophotometer. The OR (optical rotation) was recorded on a JASCO (Japan) P1020 Polarimeter equipped with a tungsten lamp (MeOH as solvent) and CD (circular dichroism) data was recorded on a JASCO (Japan) J-815 spectrometer equipped with a tungsten lamp (MeOH as solvent). Mass spectra were obtained using LCMS-IT-TOF, Shimadzu spectrometer Series Mass Selective Detector, Agilent Technologies 6530 Accurate-Mass Q-TOF LC/MS, with ZORBAX Eclipse XDB-C18 Rapid Resolution HT 4.6 mm i.d. × 50 mm × 1.8 μm column. Solvent used was methanol (CH_3_OH). The Auto mass Multi Thermofinnigan was used for HR ESI analysis and EIMS spectra were obtained on Shimadzu LCMS-IT-TOF Mass Spectrometer, QP2000A spectrometer 70 eV. NMR spectra were recorded in deuterated chloroform (CDCl_3_) and deuterated methanol CD_3_OD) on JEOL LA400 FT-NMR and JEOL ECA 400 FT-NMR as a JEOL JNM-FX400 (400 MHz for ^1^H and 100 MHz for ^13^C; unless stated otherwise) and signal of spectra calibrated using TMS. Chemical shifts were reported in ppm on δ scale, and the coupling constants were measured in Hertz (Hz). Silica gel 60, 70-230 mesh ASTM (Merck 7734) was used for various column chromatography methods such as CLC and FLC.TLC aluminum sheets and PTLC (20 × 20 cm Silica gel 60 F_254_) were used in the thin layer chromatography analysis. The TLC and PTLC spots were visualized under UV light (254 and 366 nm) followed by spraying with Dragendorff’s reagent for detection of alkaloids. Dragendorff’s reagent and Mayer’s reagent were used for alkaloid screening. All solvents used were of AR grade except those used for bulk extraction.

### 3.2. Plant Materials

The bark of *Beilschmiedia kunstleri* Gamble (Lauraceae) was collected at Hutan Simpan Sungai Tekam, Jerantut, Pahang, Malaysia by the phytochemical group of the Department of Chemistry, University of Malaya. The voucher specimen (KL5627) of this plant has been deposited at the Herbarium of the Department of Chemistry, University of Malaya, Kuala Lumpur, Malaysia.

### 3.3. Extraction and Isolation of Chemical Constituents

Dried, ground stem bark of *Beilschmiedia kunstleri *Gamble (1.50 kg) was first defatted with hexane (5.0 L) for 72 h and then the hexane extract was filtered. The residual bark material was air-dried and moistened with 15% NH_4_OH. It was then extracted with CH_2_Cl_2_ (10.0 L) for 5 days. After filtration, the supernatant was concentrated using rotary evaporator under reduced pressure to a volume of 500 mL, followed by acidic extraction with 5% HCl until a negative Mayer’s test result was obtained. The hydrochloric acid portion was washed with CH_2_Cl_2_, basified with NH_4_OH to pH 11 and re-extracted with CH_2_Cl_2_. The CH_2_Cl_2_ extract was washed with distilled H_2_O, dried over anhydrous sodium sulphate, filtered and evaporated to give crude alkaloid (2.90 g). The extraction of alkaloids was repeated by using MeOH as the solvent and crude alkaloid (10.00 g) was obtained. The crude alkaloid (2.90 g) was subjected to exhaustive column chromatography over silica gel using CH_2_Cl_2_ and gradually increasing the polarity with methanol to give 12 fractions. Fraction 9 gave a new compound **1** (38.05 mg, 1.30%, PTLC Merck KGaA silica gel 60 F_254_; CH_2_Cl_2_-MeOH; 90:10), fraction 10 gave alkaloid **3** (23.55 mg, 0.80%, CH_2_Cl_2_-MeOH; 85:15), fraction 7 afforded alkaloid **4** (19.52 mg, 0.67%, CH_2_Cl_2_-MeOH; 95:5), fraction 3 gave alkaloid **5** (19.00 mg, 0.65%, CH_2_Cl_2_-MeOH; 98:2), fraction 1 gave alkaloid **6** (32.20 mg, 1.13%, CH_2_Cl_2_-MeOH; 98:2), fraction 6 afforded alkaloid **8** (24.80 mg, 0.85%, CH_2_Cl_2_-MeOH; 96:4) and fraction 11 gave alkaloid **9** (27.21 mg, 0.93%, CH_2_Cl_2_-MeOH; 80:20).

The crude methanol extract (10.00 g) was subjected to column chromatography over silica gel to yield five fractions. Fraction 3 gave compound **2** (24.45 mg, 0.83%, CH_2_Cl_2_-MeOH; 50:50), fraction 2 afforded alkaloid **5** (29.50 mg, 1%, CH_2_Cl_2_-MeOH; 60:40) and fraction 1 gave alkaloid **7** (32.20 mg, 1.13%, CH_2_Cl_2_-MeOH; 98:2).

(−)-Kunstleramide (**1**) with [α^25^_D_]: = −15.48° (*C *= 4.2 × 10^−2^ M, MeOH), was obtained as a yellowish amorphous solid; UV (MeOH) λ_max_: 210 and 262 nm [14,15]; CD (MeOH) λ_max_ 260 (Δε + 4.0), 265 (Δε −3.5); (IR bands (KBr): 3,351.5, 1,659.4, 1,612.2, 351.5 cm^ −1^[15,16]; ^1^H-NMR (400 MHz, CDCl_3_) and ^13^C-NMR (100 MHz, CDCl_3_): (Table 1); LC-MS, MHz*: *328.1531 [M+Na]*^+ ^*(calc. 328.1525 for C_17_H_23_NO_4_).

### 3.4. Antioxidant Assay

The DPPH assay was performed according to the method reported by Orhan *et al*. [22] and Brem *et al*. [23], with modifications. Briefly, 0.02% stable DPPH free radical (50 µL) in methanol (100 mL) was added to standard/sample/control (20 µL) and methanol (130 µL, total assay volume 200 µL) in a 96-well plate. Ascorbic Acid (vitamin C) was used as the standard and blank solvent methanol as the negative control. The absorbance was read at 517 nm using SUNRISE Microplate Absorbance Reader after 30 min of incubation at room temperature. The percentage of DPPH free radical inhibition activity was determined according to the formula: 


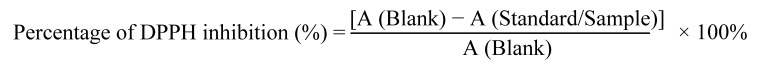


where A (Blank) refers to the absorbance of the blank solvent and DPPH at 517 nm while A(Standard/Sample) refers to the absorbance of Ascorbic Acid and the samples at 517 nm. This formula was also used to determine the concentration of each sample required to quench 50% of the DPPH free radical activity (IC_50_ value) [24].

### 3.5. Statistical Analyses

Each experiment was performed at least twice. Results are expressed as the means value ± standard deviation (SD). Log IC_50_ calculations were performed using the built-in algorithms for dose-response curves with variable slope using Graphpad Prism software (version 4.0; GraphPad Software Inc., San Diego, CA, USA). A fixed maximum value of the dose-response curve was set to the maximum obtained value for each drug.

### 3.5. Cytotoxic Activity Studies

#### 3.5.1. Cell Culture

All the cells used in this study were obtained from American Type Cell Collection (ATCC) and maintained in a 37 °C incubator with 5% CO_2_ saturation. A375 human melanoma, HT-29 human colon adenocarcinoma cells and WRL-68 normal hepatic cells were maintained in Dulbecco’s modified Eagle’s medium (DMEM), whereas A549 non-small cell lung cancer cells and PC-3 prostate adenocarcinoma cells were maintained in RPMI medium. Both medium were supplemented with 10% fetus calf serum (FCS), 100 units/mL penicillin, and 0.1 mg/mL streptomycin.

#### 3.5.2. Cellular Viability

Different cell types mentioned above were used to evaluate the inhibitory effect of kunstleramide (**1**) on cell growth using the MTT assay. The MTT assay was modified as described by Cheah *et al*. and Mosmann [25,26]. Briefly, cells were seeded at a density of 1 × 10^5^ cells/mL in a 96-well plate and incubated for 24 h at 37 °C, 5% CO_2_. The next day, cells were treated with the compounds respectively and incubated for another 24 h. After 24 h, MTT solution at 2 mg/mL was added and incubatefor 1 h. Absorbance at 570 nm was measured and recorded using a Plate Chameleon V microplate reader (Hidex, Turku, Finland). Results were expressed as a percentage of control giving percentage cell viability after 24 h exposure to test agent. The potency of cell growth inhibition for each test agent was expressed as an EC_50_ value, defined as the concentration that caused a 50% loss of cell growth. Viability was defined as the ratio (expressed as a percentage) of absorbance of treated cells to untreated cells [27].

## 4. Conclusions

To the knowledge of the authors, (−)-kunstleramide (**1**) is the first ethyl dienamide reported in the family of Lauraceae which bears a hydroxyl group at C-6 and two methoxyl groups attached to C-3' and C-4'. This is the first communication on a dienamide from *Beilschmiedia kunstleri*. The neolognan (+)-kunstlerone (**2**) and alkaloids **3**-**9**, belonging to the aporphine, benzylisoquinoline, morphinandienone and pavine type of alkaloids were also isolated from this plant. (−)-Kunstleramide (**1**) exhibited very poor DPPH, activity with an IC_50_ value of 179.5 ± 4.4 μg/mL compared with the DPPH inhibitor ascorbic acid. Kunstleramide (**1**) showed moderate cytotoxic effect with EC_50_ values. This study revealed that this plant showed promising cytotoxic activity but poor DPPH activity. Further investigation should be carried out to evaluate the cytotoxicity of compound **1** at lower concentrations and its mechanism.

## Supplementary Materials

Supplementary materials can be found at: http://www.mdpi.com/1420-3049/17/4/4197/s1.
