# Supplementary material for: (−)-Kunstleramide, a New Antioxidant and Cytotoxic Dienamide from the Bark of Beilschmiedia kunstleri Gamble
Source: Molecules. 2012 Apr 5;17(4):4197–208. doi: 10.3390/molecules17044197 (PMC6268565; doi:10.3390/molecules17044197)

Supplementary Material

Figure 1S. IR spectrum of (−)-*kunstleramide* (1).

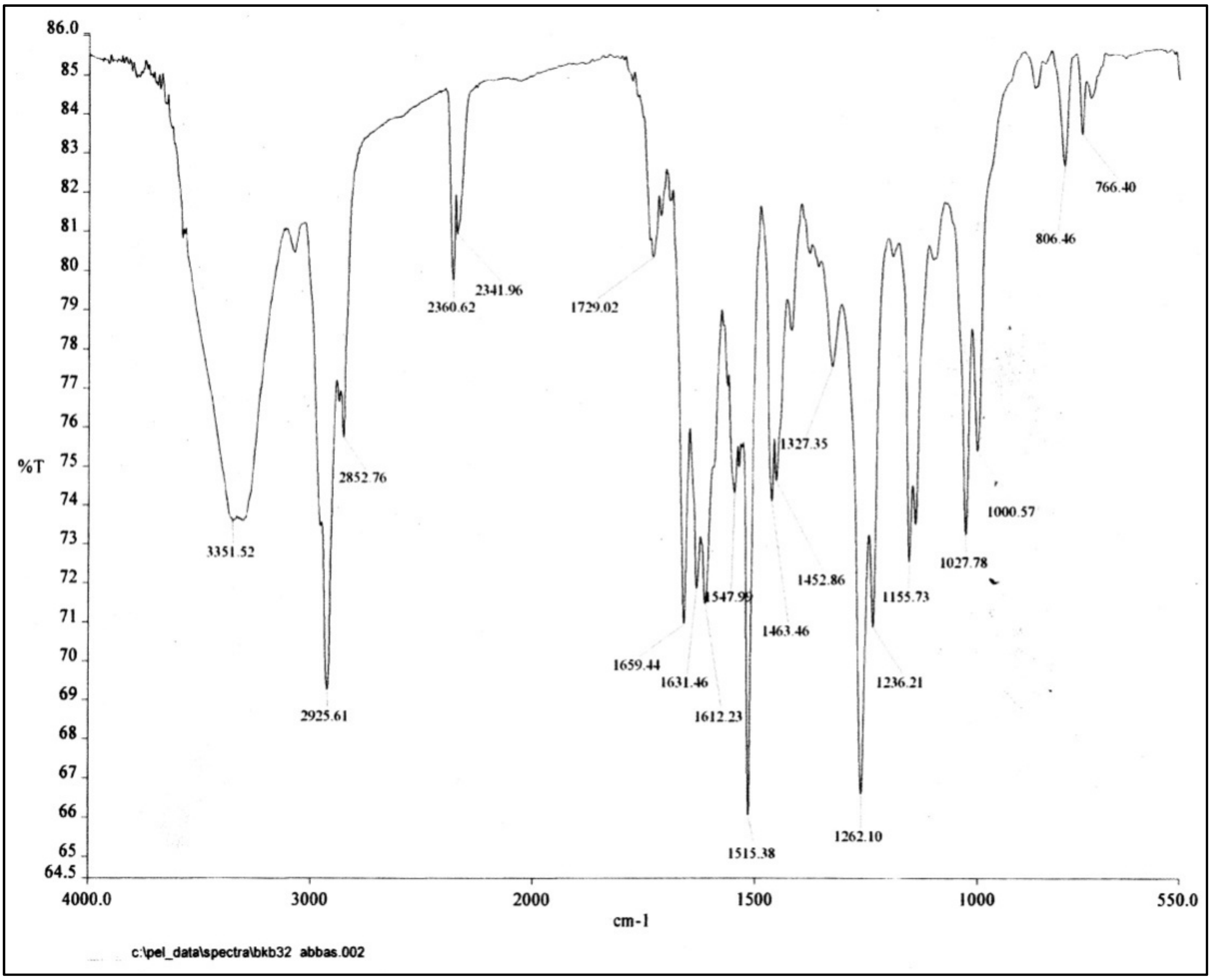

Figure 2S. CD spectrum of (−)-*kunstleramide* (1).

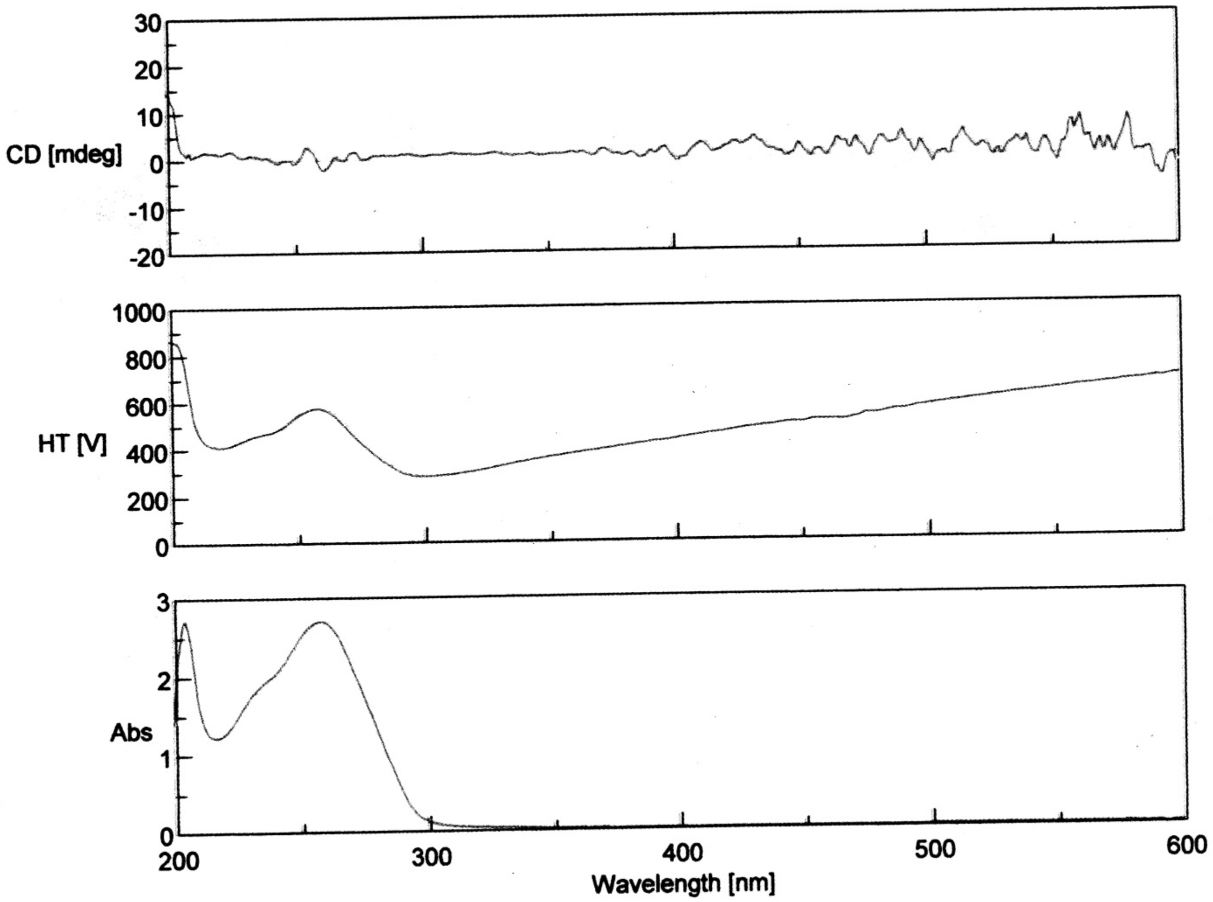

Figure 3S. MS of (−)-*kunstleramide* (1).

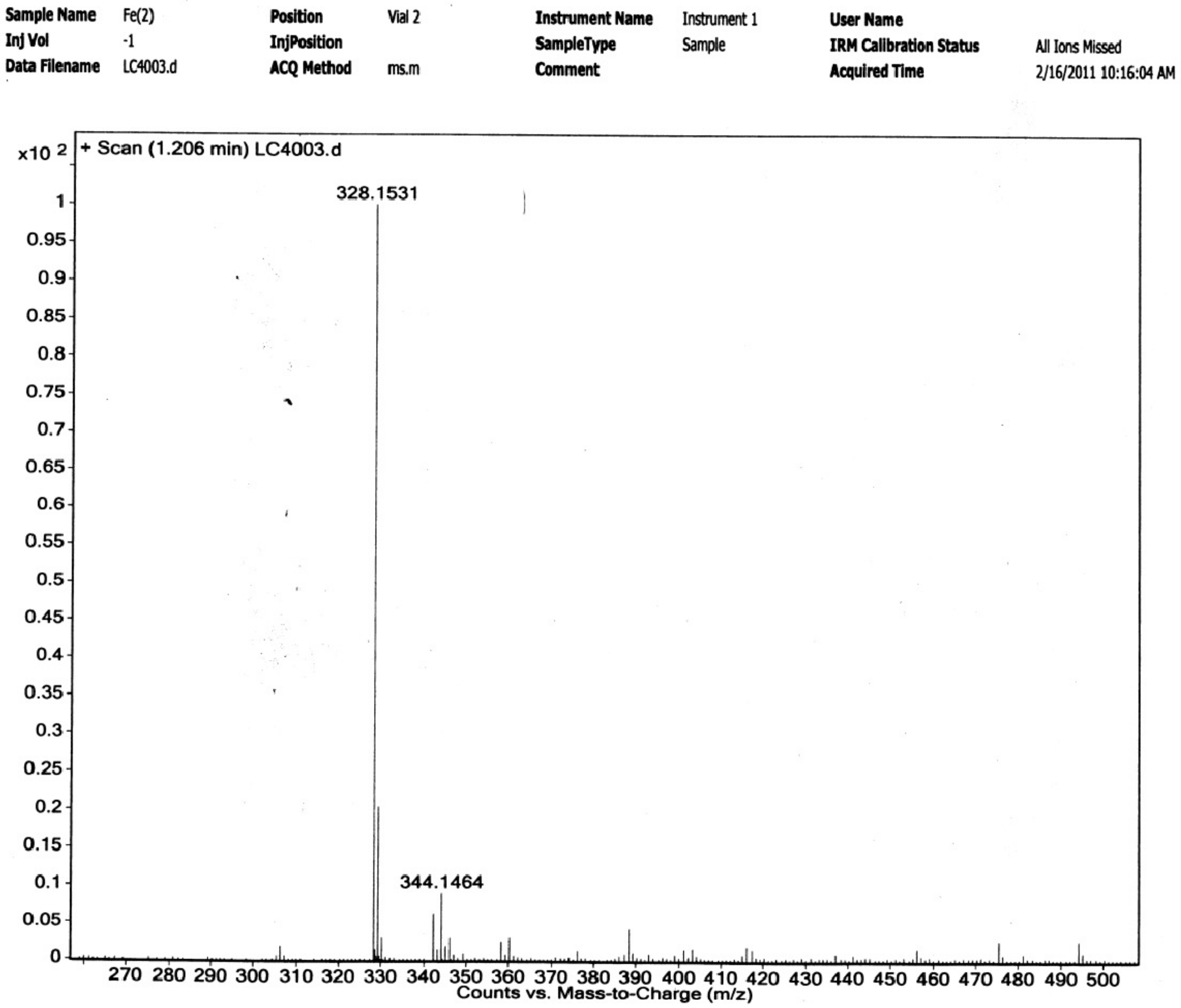

Figure 4S. ^{1}H-NMR spectrum of (−)-*kunstleramide* (1).

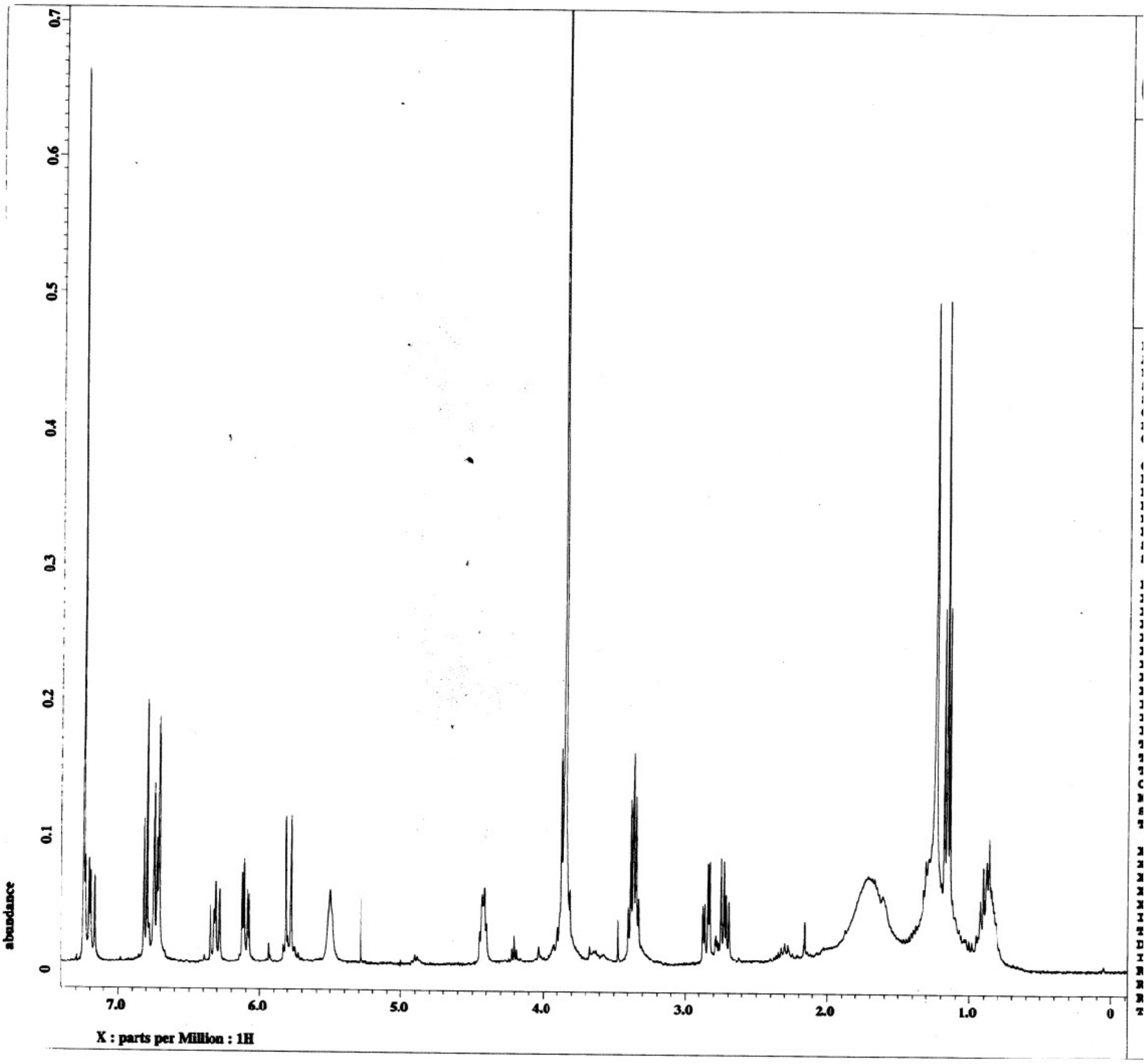

Figure 5S. ^{13}C-NMR spectrum of (−)-*kunstleramide* (1).

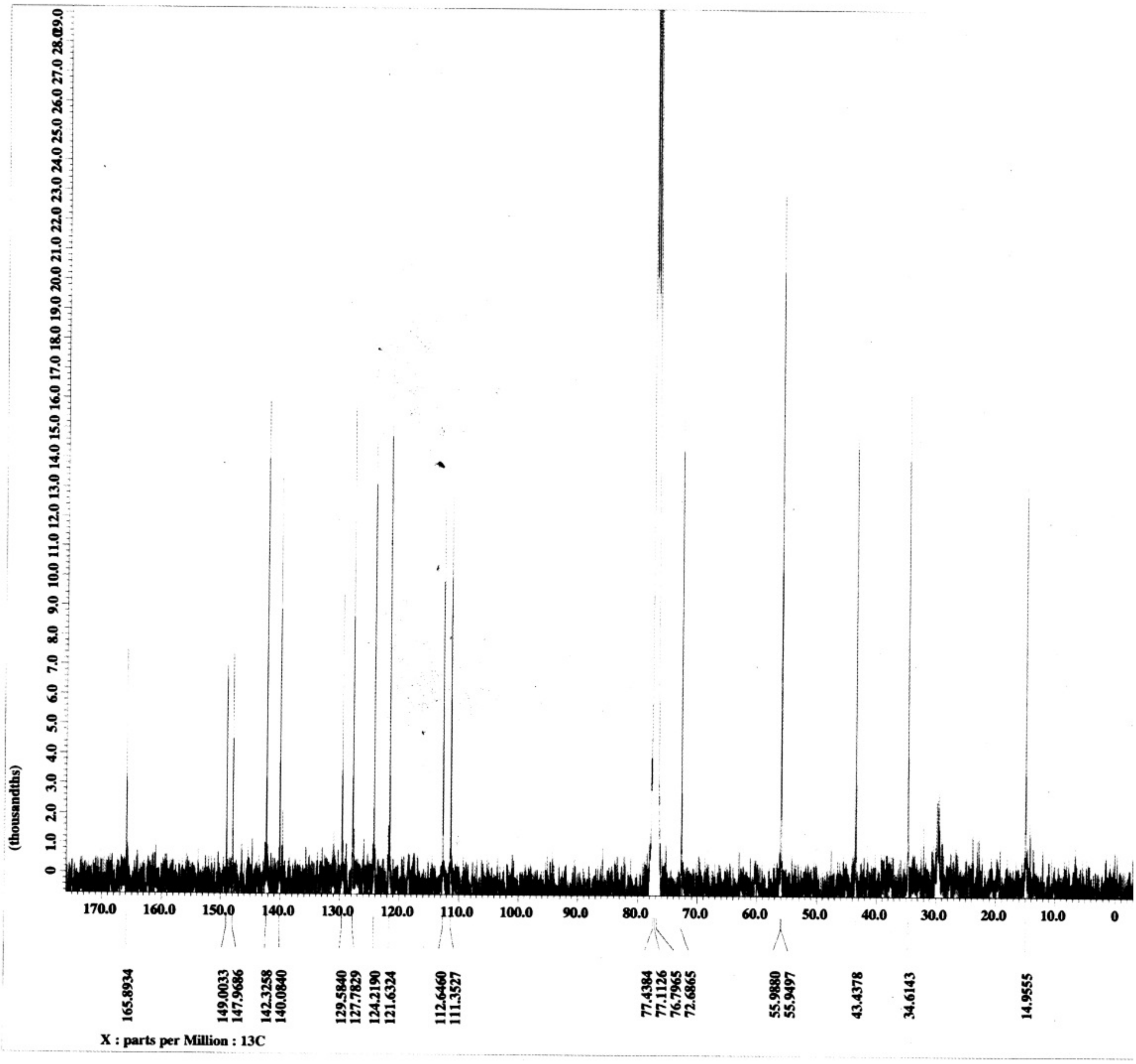

Figure 6S. DEPT of (−)-*kunstleramide* (1).

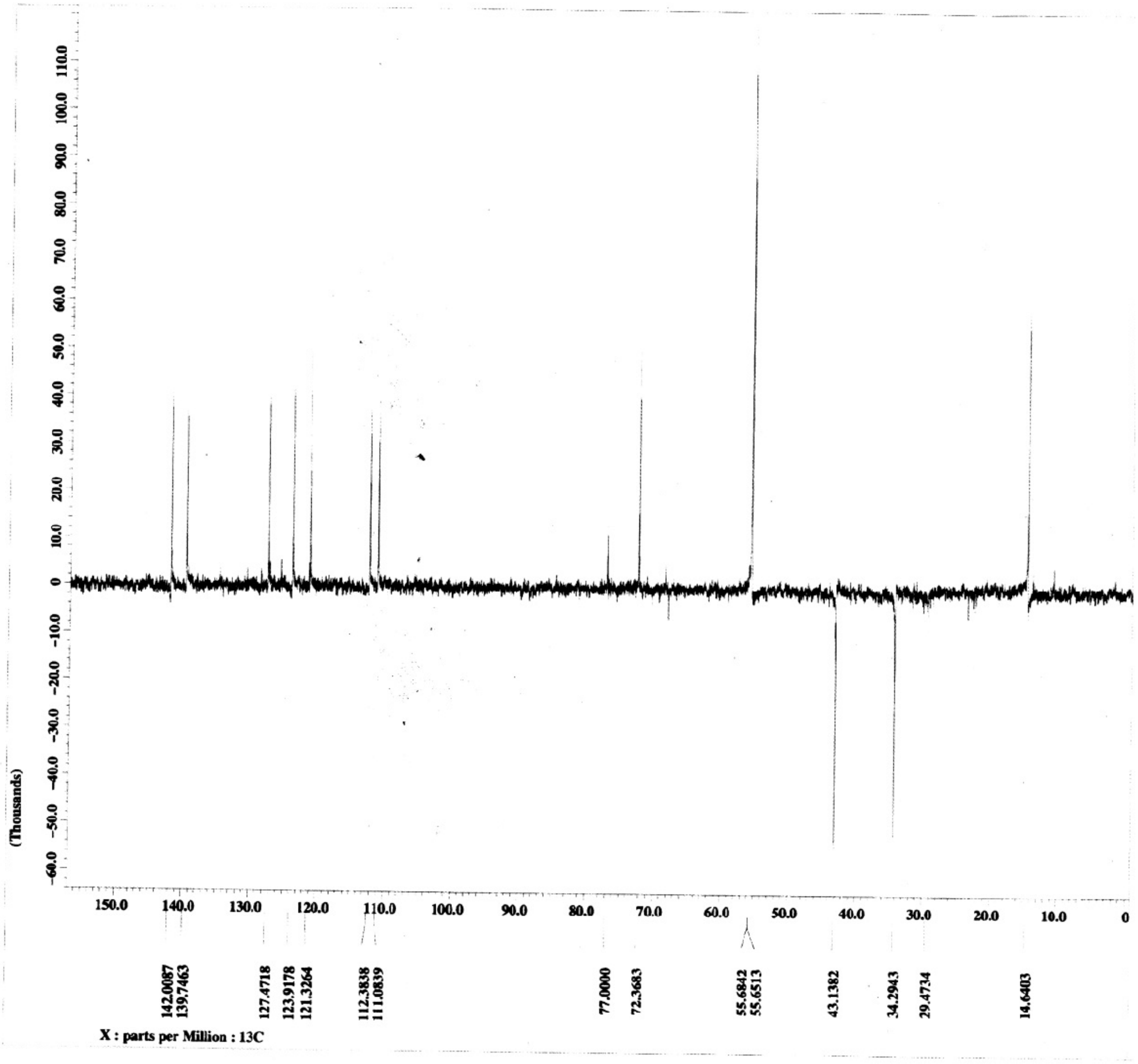

Figure 7S. COSY spectrum of (−)-*kunstleramide* (1).

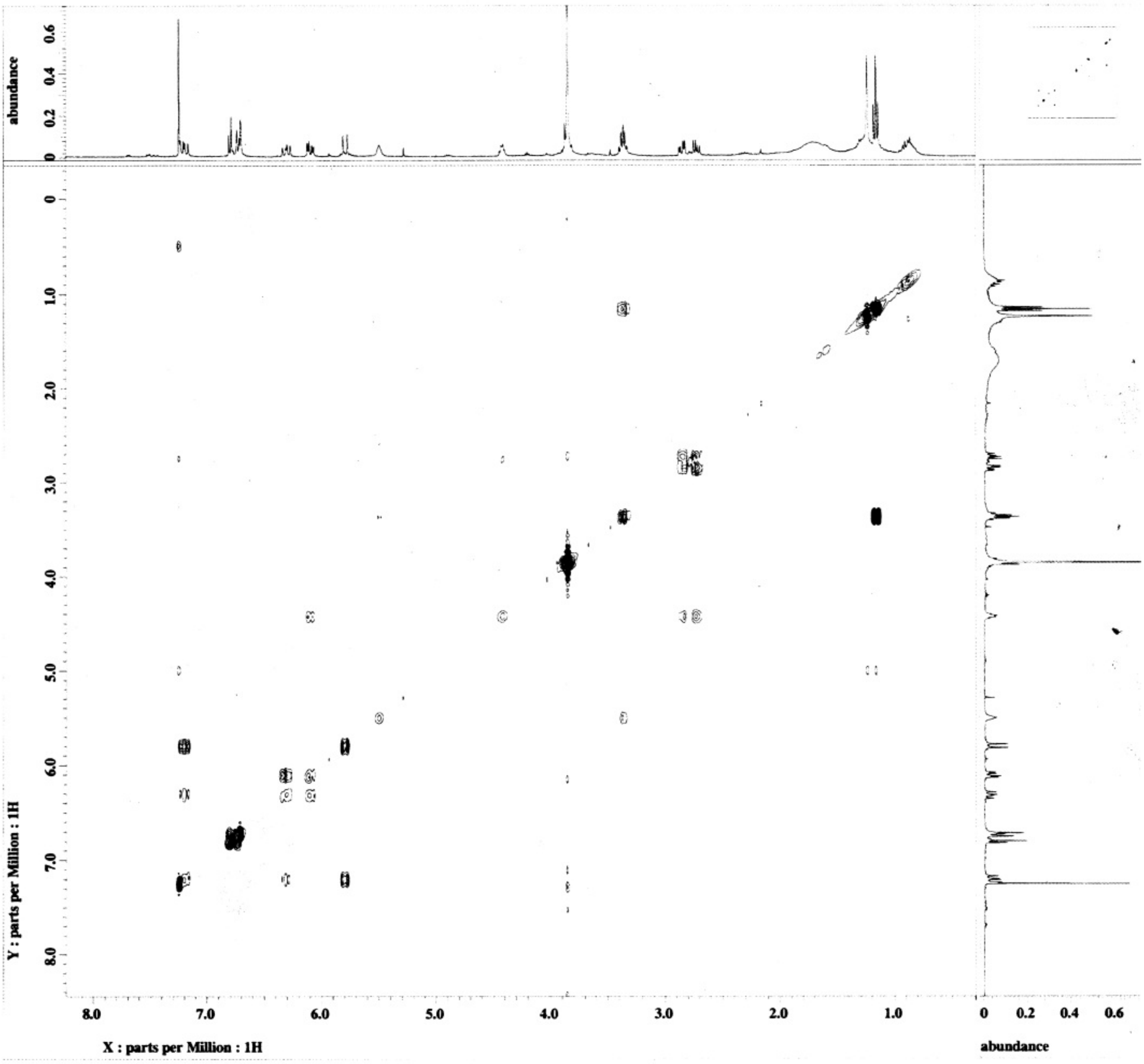

Figure 8S. NOESY spectrum of (−)-*kunstleramide* (1).

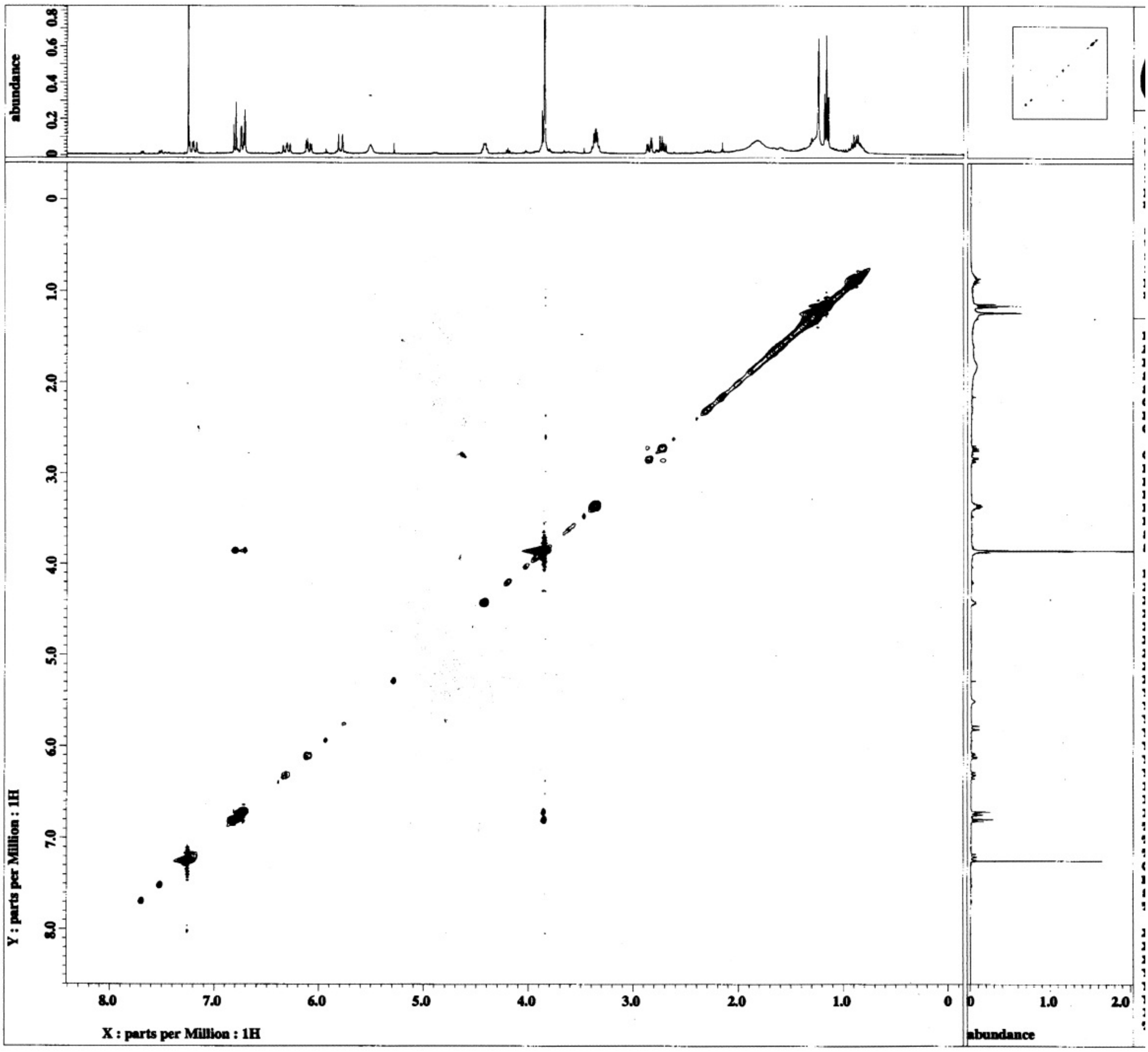

Figure 9S. HMQC spectrum of (−)-*kunstleramide* (1).

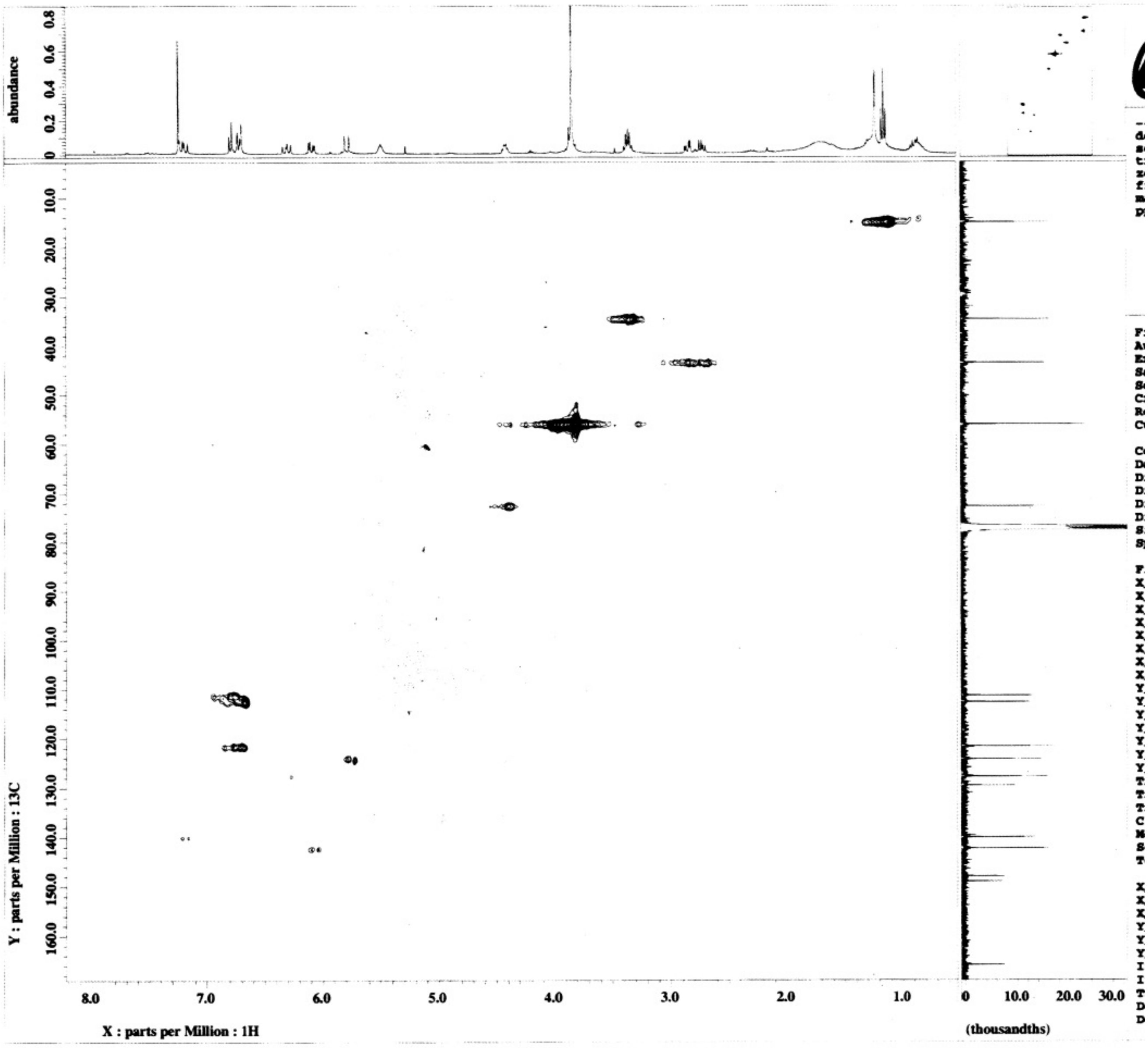

Figure 10S. HMBC spectrum of (−)-*kunstleramide* (1).

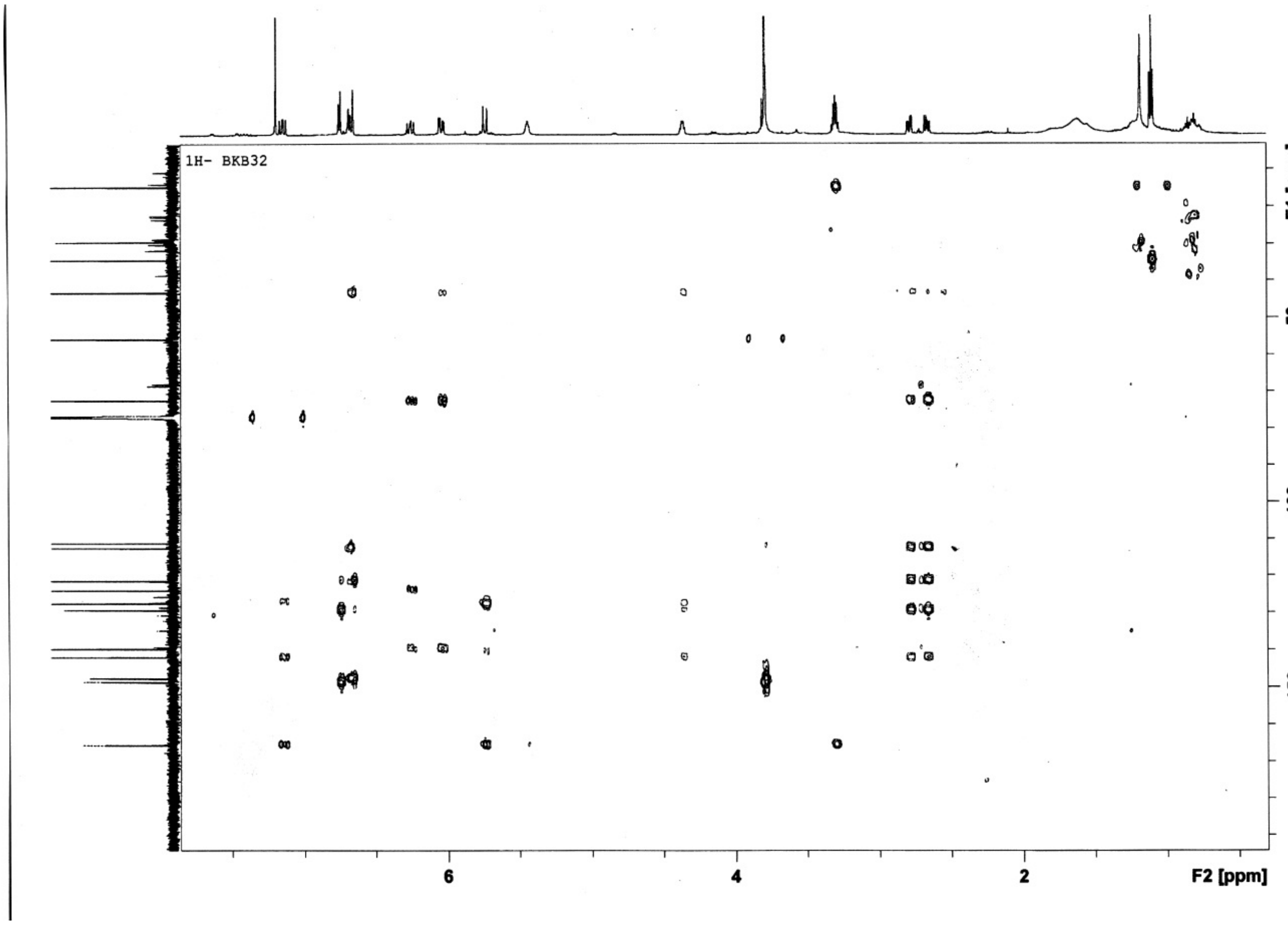

Supplement: Supplementary file 1 [file molecules-17-04197-s001.pdf]
